# Effects of Sc and Zr Addition on Microstructure and Mechanical Properties of AA5182

**DOI:** 10.3390/ma14164753

**Published:** 2021-08-23

**Authors:** Jingxiao Li, Xiaofang Yang, Shihua Xiang, Yongfa Zhang, Jie Shi, Youcai Qiu, Robert Edward Sanders

**Affiliations:** 1 Shenyang National Laboratory for Materials Science, Chongqing University, Chongqing 400044, China; lijingxiao@cqu.edu.cn (J.L.); xiangsh@cqu.edu.cn (S.X.); qiuyoucaicqu@foxmail.com (Y.Q.); 2 International Joint Laboratory for Light Alloys (Ministry of Education), College of Materials Science and Engineering, Chongqing University, Chongqing 400044, China; yongfa.zhang@iehk.rwth-aachen.de (Y.Z.); 13308375814@163.com (J.S.)

**Keywords:** AA5182, recrystallization, strength, Sc and Zr microalloying

## Abstract

The effects of 0.1 wt.% Sc and 0.1 wt.% Zr addition in AA5182 on microstructure and mechanical properties were investigated. Results show that Al_3_(Sc_x_Zr_1−x_) dispersoids formed in AA5182. Observation of ingots microstructures showed that the grain size of 5182-Sc-Zr alloy was 56% lower than that of based AA5182. Isothermal annealing between 230 °C and 500 °C for 2 h was performed to study the recrystallization, tensile properties and dispersoid coarsening. The recrystallization was inhibited by the dispersoids, and the alloy microstructure remained deformed after annealing. Al_3_(Sc_x_Zr_1−x_) in AA5182 was stable when annealing below 400 °C, while parts of dispersoids coarsened significantly when heating at 500 °C. The addition of Sc and Zr allowed YS of 5182 alloy to achieve 247.8 MPa, which is 100 MPa higher than the corresponding AA5182. The contributions of Orowan strengthening and grain boundary strengthening were obtained by calculation.

## 1. Introduction

Aluminum microalloying with rare earth elements has been a burning subject in recent years. Commonly added elements include Sc and Er, which can form nanometric Al_3_X (X = Sc or Er) precipitates in aluminum alloys [1]. Because Er has a higher diffusion coefficient than Sc and the Al_3_Er particles are the less resistant to coarsening during heating, most studies have favored the addition of scandium to improve performance. The addition of Sc to Al alloys can significantly improve mechanical properties of Al alloys by forming a stable Sc containing dispersoids and inhibiting recrystallization [1,2,3,4,5]. As shown by Filatov [6], the addition of Sc to the Al-Mg system alloy results in grain refinement, dendrites elimination and additional strengthening. For purposes of grain refinement and strength enhancement, the added amount of Sc needs to be higher than 0.55% (eutectic composition) [7]. Teng’s study shows that the grain size of AA7055 was reduced from 30 μm to 10 μm with the addition of 0.25% Sc, demonstrating that in multicomponent Al alloys grain refinement can still be achieved with a lower addition of Sc than the eutectic composition [8]. The addition of a small amount of Sc to a multicomponent Al alloy improves the performance of the alloy while reducing the impact of the high price of Sc on the cost of the alloy. In the current research, a composite addition of Sc and Zr is often utilized to reinforce Al alloy. For Al-Sc-Zr alloys, after homogenization treatment, a Sc- and Zr-bearing dispersoid is formed, which is a bilayer structure consisting of a Zr-rich shell and a Sc-rich core [9]. Since the diffusion coefficient of Zr in the Al matrix is lower than that of Sc, replacing Sc with Zr in the shell increases the coarsening resistance of Al_3_(Sc_x_Zr_1−x_) dispersoids [9,10,11,12,13,14]. It is widely accepted that the Sc- and Zr-bearing dispersoids can effectively improve the mechanical properties of Al alloy by inhibiting the motion of dislocations and grain boundaries. It has been shown that the composite addition of Sc and Zr brings remarkable strengthening effects to Al-Zn-Mg alloys [15]. After annealing at high temperature, the Sc- and Zr-bearing particles (Al_3_(Sc_x_Zr_1−x_) dispersoids) prevent recrystallization by inhibiting movement of grain boundaries and dislocations, so that the recrystallization temperature of the Al alloy almost reaches the solidus temperature, which effectively improves the recrystallization resistance of the alloy [16]. In Tang’s research [17], by dispersoid strengthening, the yield strength of Al-Mg-Sc-Zr alloy was 480 MPa, which was an increase of 60 MPa more than Al-Mg alloy.

An increasing number of reports in the literature have investigated the influence of Sc and Zr on aluminum alloys; most research has been focused on studying the changes and influence of dispersoids in binary or ternary aluminum alloys. For example, the study of Yin shows that adding 0.2% Sc and 0.1% Zr to as-hot rolled Al-5Mg alloy increased the strength of the alloy by 150 MPa [5]. A limited literature exists on the addition of Sc and Zr to commercial Al alloys. As a promising potential structural material, 5182 alloy stands out by its good corrosion resistance, weldability and formability. In order to expand the application of 5182 alloy, improving its strength is of foremost importance. Microalloying 5182 alloy by Sc or Zr is one of the effective approaches for enhancing its mechanical properties [7] that has been less studied. The main strengthening mechanism in 5182 alloy is the solid solution strengthening brought by Mg and Mn [4]. There are few studies on the substantial strength enhancement to 5182 alloy by adding small amounts of Sc-Zr. In this study, reinforcement was expected by microalloying with a small amount of Sc and Zr. The aim of this work was to study the effects of Sc and Zr microalloying on the as-cast microstructure of 5182 alloy, the recrystallization microstructure and the precipitation strengthening of the alloy after cold rolling. The effect of Sc and Zr on the microstructure of 5182 alloy was observed by optical microscope (OM), electron back scattering diffraction (EBSD) and transmission electron microscopy (TEM). The mechanical properties of the 5182-Sc-Zr alloy were compared with those of the 5182 alloy after annealing, and the strengthening contribution of Al_3_(Sc_x_Zr_1−x_) phase was calculated.

## 2. Experimental Procedure

The as-cast materials used in this experiment were provided by Clean TeQ. The average composition of alloys, which were measured by optical emission spectroscopy, are listed in Table 1. In this study, two kinds of alloys were investigated: one was AA5182 referenced alloy, the other one was 5182 alloy containing 0.1wt.% Sc and 0.1 wt.% Zr (referred to as 5182-Sc-Zr alloy hereafter).

The surface of ingots were milled by sandpapers in an order of 120#, 240#, 400#, 800#, 1000#, 1500#, 2000#, 3000#, 4000#. A 60 °C 10% NaOH solution and 50% HNO_3_ were applied to etch the surface of the ingots. In this study, the parameters of two-stage isothermal homogenization were 275 °C for 20 h and 440 °C for 12 h. Samples after homogenization were subjected to multiple passes of hot rolling and cold rolling. The heating temperature of hot rolling was 510 °C, and the ingots were kept in the furnace for 40 min before rolling and were finally rolled to 10 mm. After multiple passes of cold rolling, sheets with a cold rolling reduction rate of 70% and a thickness of 3 mm were obtained.

The cold rolled sheets were annealed at 230 °C, 250 °C, 300 °C, 400 °C and 500 °C for 2 h, followed by water quenching, respectively. The longitudinal sections of the annealed sheets were polished by sandpapers. An amount of 10% perchloric acid reagent and 90% absolute ethanol solution was used to polish the annealed samples. EBSD data were collected on a Zeiss FIB/SEM at 20 Kv with a step of 0.5 μm to calibrate the longitudinal sections of annealed material, and recrystallization degree of the material was confirmed by Channel 5 software. Transmission electron microscopy (TEM) samples were prepared by twin-jet polishing and then examined using a FEI Talos F200S electron microscope operating at 200 kV. The [110] axis of Al matrix was selected for dark field observation. Tensile tests were conducted on the cold rolled and as-annealed sheets along the rolling direction, which were performed by a Shimadzu AGX (50 KN) machine with a strain rate of 1 mm/min. The strain was measured using a calibrated linear variable differential transformer attached to an optical automatic extensometer. The stress–strain data were collected through a computerized data acquisition system. All of the specimens were tested to failure.

## 3. Results

### 3.1. Effect of Sc and Zr Microalloying Additions on Microstructure

**Figure 1:**
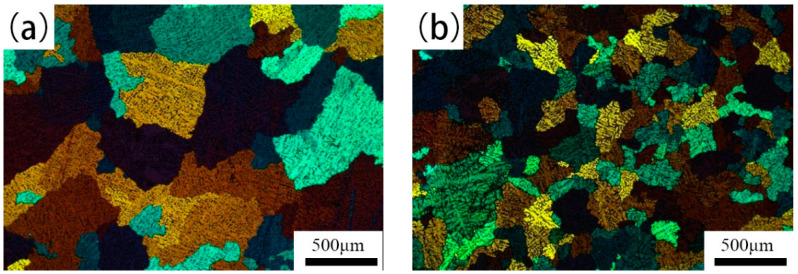
Microstructure of as-cast samples: (**a**) 5182 alloy; (**b**) 5185-Sc-Zr alloy.

The microstructures of the ingots can be observed in Figure 1. The average grain size of the 5182-Sc-Zr alloy was about 0.4 mm (±0.2 mm), whereas the average grain size of the 5182 alloy was 0.7 mm (±0.4 mm). The difference of the grain size showed that the ingot microstructure was significantly refined after adding 0.1% Sc and 0.1% Zr. According to Zhou et al. [18], primary Al_3_(Sc_x_Zr_1−x_) precipitated in the casting process, which could promote nucleation and lead to grain refinement.

**Figure 2:**
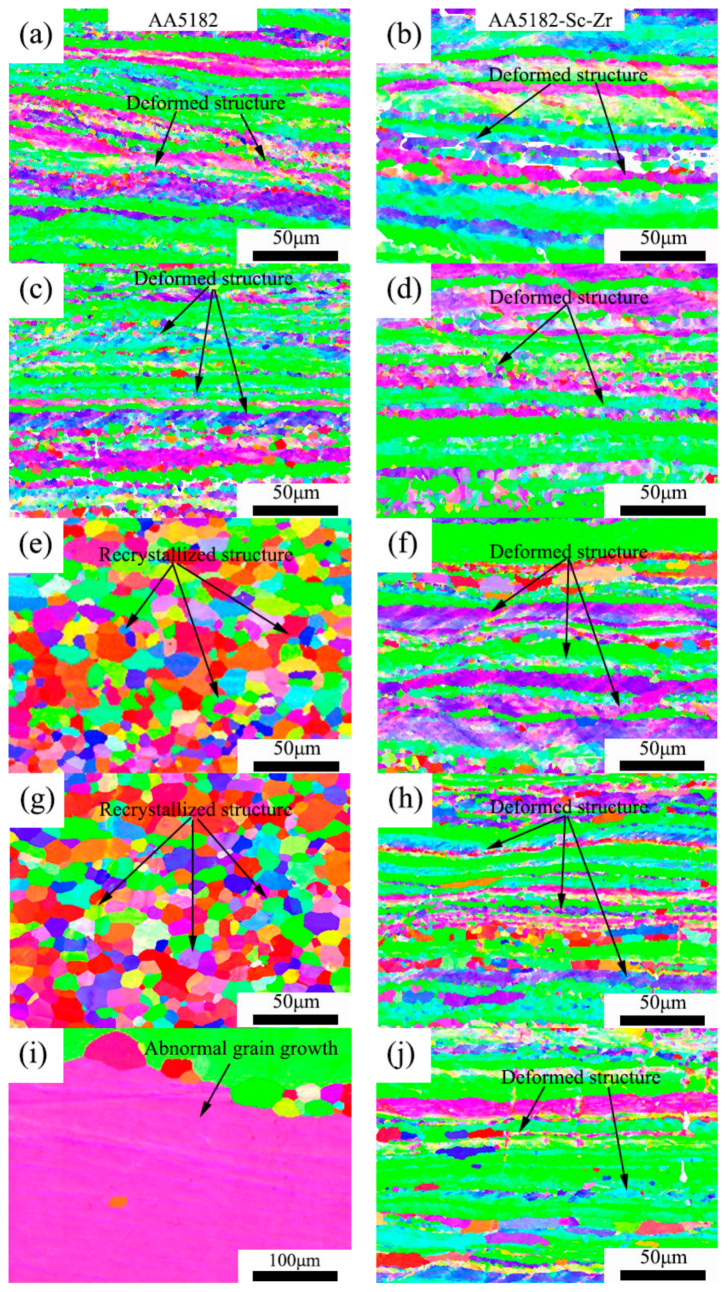
Microstructures after isothermal annealing for 2 h: (**a**) 5182 alloy annealed at 230 °C; (**b**) 5182-Sc-Zr alloy annealed at 230 °C; (**c**) 5182 alloy annealed at 250 °C; (**d**) 5182-Sc-Zr alloy annealed at 250 °C; (**e**) 5182 alloy annealed at 300 °C; (**f**) 5182-Sc-Zr alloy annealed at 300 °C; (**g**) 5182 alloy annealed at 400 °C; (**h**) 5182-Sc-Zr alloy annealed at 400 °C; (**i**) 5182 alloy annealed at 500 °C; (**j**) 5182-Sc-Zr alloy annealed at 500 °C.

Microstructures of samples that were rolled to a strain of 70% and were annealed at 230 °C, 250 °C, 300 °C, 400 °C, 500 °C for 2 h were studied by EBSD and are shown in Figure 2. The recrystallization rate of the annealed samples was analyzed by Channel 5, where the recrystallization fraction was determined by measuring the average internal misorientation angle of the grains. In this study, the grains whose internal misorientation were above 10° were classed as recrystallized. The results are shown in Table 2. After annealing at 230 °C and 250 °C, 5182 alloys were partially recrystallized, and the recrystallization rates were 11.8% and 18.9%, respectively, which were slightly higher than those of the 5182-Sc-Zr alloy (10.3% and 10.8%). After annealing at 300 °C and 400 °C for 2 h, the recrystallization rates of 5182 alloys were both higher than 90%, while the recrystallization degree of 0.1% Sc-containing alloy was 15.7% and 24.4%, respectively. After annealing at 500 °C, part of the grains in the 5182 alloy grew abnormally (Figure 2i), and the recrystallization degree of the 5182-Sc-Zr alloy was 30.1%. As can be seen, recrystallization of the 5182-Sc-Zr alloy was impeded during the annealing process. The result indicates that the recrystallization rate of the 5182-Sc-Zr alloy was significantly lower than that of the 5182 under the same condition, which means the movement of grain boundaries in the Al-Mg-Sc-Zr alloy during the recrystallization process was hindered by Al_3_(Sc_x_Zr_1−x_) [19].

**Figure 3:**
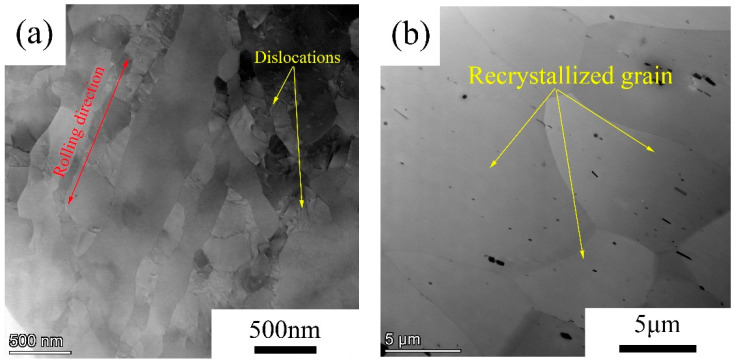
TEM of 5182 alloy: (**a**) annealed at 250 °C; (**b**) annealed at 300 °C.

The microstructures of annealed alloys were further studied by TEM. Figure 3 shows the bright field microstructure of 5182 alloys. In Figure 3a, after annealing at 250 °C for 2 h, a large number of dislocations remained in the 5182 alloy. In combination with Figure 1, it can be concluded that partial recrystallization occurred in the 5182 alloy after annealing at 250 °C. Figure 3b shows the microstructure of the 5182 alloy after annealing at 300 °C. In Figure 3b, no dislocation was observed in the grains of 5182 alloy. The combination of microstructure and the recrystallization rate determined that alloy 5182 was completely recrystallized.

**Figure 4:**
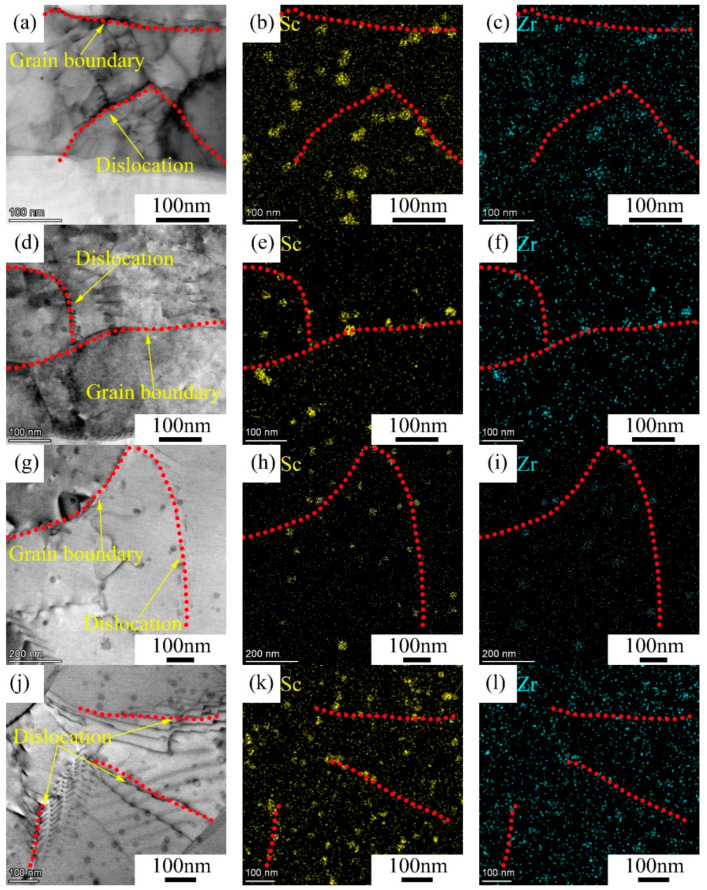
TEM of annealed 5182-Sc-Zr alloy: (**a**–c) annealed at 250 °C; (**d**–**f**) annealed at 300 °C; (**g**–**i**) annealed at 400 °C; (**j**–**l**) annealed at 500 °C.

Figure 4 shows the bright field and the corresponding EDS of the 5182-Sc-Zr alloy after annealing. In Figure 4a,d, it can be seen that a large number of dislocations were retained after annealing at 250 °C and 300 °C. Figure 4a–c shows the bright field of the corresponding EDS of samples annealed at 250 °C. The dispersoids were randomly distributed in the grains. According to the EDS diagram, these dispersoids were Al_3_(Sc_x_Zr_1−x_). As Figure 4b,c shows, the dislocation piles up near the Al_3_(Sc_x_Zr_1−x_) dispersoids (as red dotted lines show). In Figure 4d–f, after annealing at 300 °C, many dislocations tangled around the dispersoids, but the dislocations disappeared in the 5182 alloy (Figure 3b). In Figure 4g,j and the corresponding EDS, after annealing at 400 °C and 500 °C, in the 5182-Sc-Zr alloy, a large number of dislocations were found near the Al_3_(Sc_x_Zr_1−x_) dispersoids. Annihilation of dislocations occurred remarkably in the 5182 alloy, while a considerable amount of dislocations piled up around dispersoids in the 5182-Sc-Zr alloy, which proves that the dispersoids play an effective role in preventing the movement of dislocations and grain boundaries. In addition, these dispersoids can pin effectively not only dislocations but also high-angle grain boundaries [20], thus preventing the 5182-Sc-Zr alloy from complete recrystallization.

**Figure 5:**
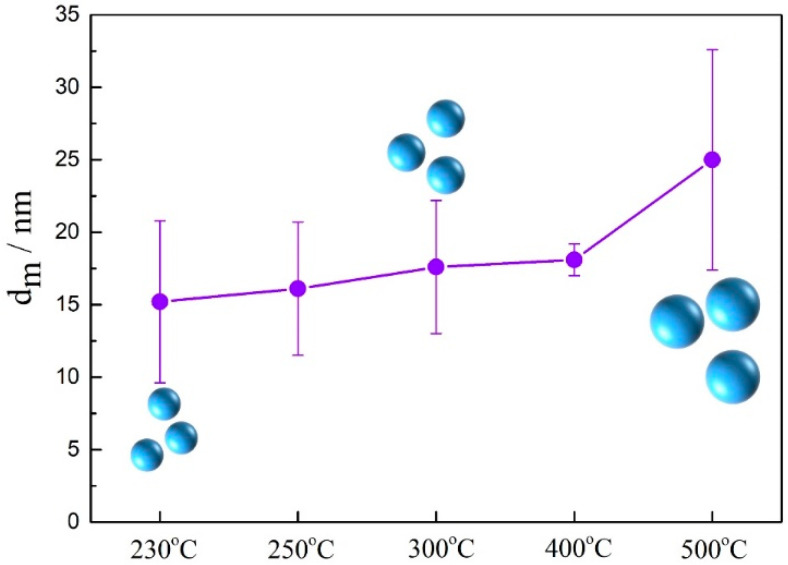
Average diameter of the Al_3_(Sc_x_Zr_1−x_) dispersoids after annealing.

The growth behavior of dispersoids was further investigated. The size change of dispersoids after annealing is shown in Figure 5. When annealed below 400 °C, the average diameter of Al_3_(Sc_x_Zr_1−x_) dispersoids was approximately 20 nm, and no large dispersoids appeared. When annealing at 500 °C for 2 h, a dispersoid with a diameter of 50 nm appeared in the 5182-Sc-Zr alloy. As the annealing temperature rose from 230 °C to 500 °C, the average size of the dispersoids changed from 15.2 ± 5.6 nm to 25 ± 7.6 nm. At 500 °C, part of the dispersoids grew up abruptly, and dispersoids 50 nm in size appeared. It can be deduced from the size change of the dispersoids after annealing that increasing the annealing temperature resulted in a coarsening of Al_3_(Sc_x_Zr_1−x_) dispersoids. In addition, Al_3_(Sc_x_Zr_1−x_) was relatively stable when annealing below 400 °C, while higher temperature led to the coarsening of dispersoids.

### 3.2. Effect of Sc and Zr Microalloying on Tensile Properties

**Figure 6:**
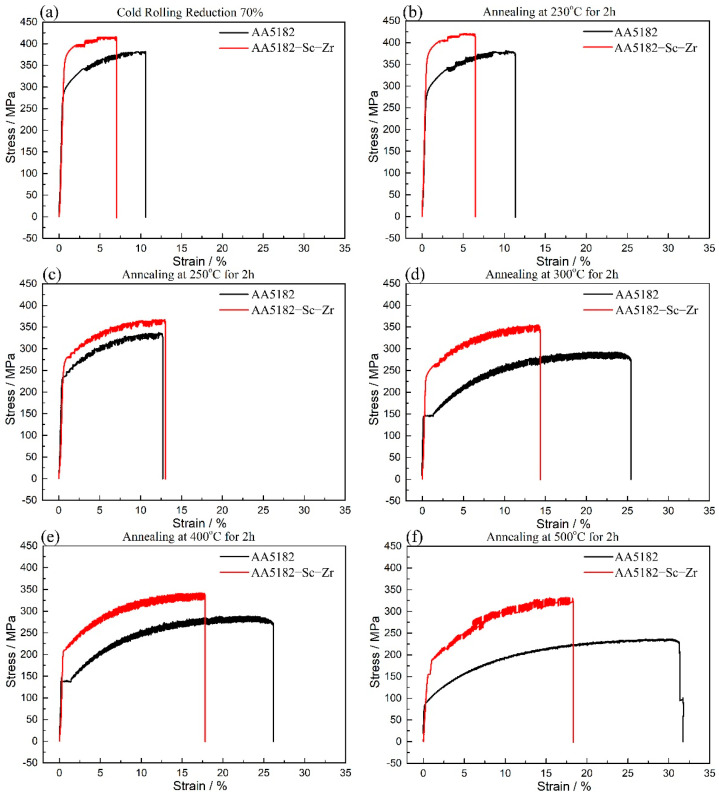
Stress–strain curves, samples with cold rolling reduction rate of 70% (**a**) cold rolled; (**b**) cold rolled and annealed at 230 °C; (**c**) cold rolled and annealed at 250 °C; (**d**) cold rolled and annealed at 300 °C; (**e**) cold rolled and annealed at 400 °C; (**f**) cold rolled and annealed at 500 °C.

Tensile tests were conducted on cold rolled sheets and annealed sheets. The stress–strain curves are illustrated in Figure 6. Table 3 lists the corresponding tensile properties of the sheets. The stress–strain curves show that the yield strengths of cold rolled 5182 alloy and 5182-Sc-Zr alloy were 283.0 MPa and 371.7 MPa, respectively. The yield strength of the AA5182 alloy decreased as the annealing temperature increased. When annealing at 400 °C, the 5182 alloy was entirely recrystallized, and its strength decreased considerably from 267.8 MPa to 147.7 MPa. When annealed at 500 °C, some grains grew up abnormally, resulting in further reduction in YS strength to 94.6 MPa. As shown in Figure 6, the yield strength of the 5182-Sc-Zr alloy varied from 371.1 MPa to 187.4 MPa when the annealing temperature increased from 230 °C to 500 °C. The yield strength of the 5182-Sc-Zr alloy annealed at 400 °C and 500 °C was 211.8 MPa and 187.4 MPa, respectively, which was 72.6 MPa and 92.8 MPa higher than those of the corresponding 5182 alloys. The resulting increase of strength indicates that adding 0.1% Sc and 0.1% Zr can improve the strength of 5182 alloy. For the change in strain-hardening exponent (n) shown in Figure 6 and Table 3, it can be seen that the strain-hardening exponent of the 5182-Sc-Zr alloy was slightly lower than that of the 5182 alloy. Theoretically, the addition of Sc and Zr reduces the uniform deformability of the alloy. However, in Chen’s study [21], it was noted that for the 5182 alloy, the effect of the strain-hardening exponent on the material forming limit was negligible, so in general, the addition of Sc and Zr brought an improvement in the mechanical properties of the 5182 alloy.

In comparison with other studies of Sc-Zr microalloying, the 5182-Sc-Zr alloy in this study was found to exhibit a high level of performance improvement. When 0.3%Sc was added to pure aluminum, the yield strength after annealing reached 209 MPa, which is 179 MPa higher than that of the pure aluminum (30 MPa) [22]. By adding 0.3% Sc and 0.1% Zr, the yield strength of Al-4.2%Mg alloy increased from 140 MPa to 280 MPa [6]. The yield strength of Al-5%Mg alloy increased by 113 MPa after adding 0.6% Sc [5]. The lower combined additions of Sc and Zr in this study still maintained a better strengthening effect compared with the alloys in the Seidman and Filatov studies. In this study, the yield strength of 5182-Sc-Zr alloys was still around 100 MPa higher than that of 5182 alloys after annealing at 230 °C, 300 °C and 500 °C. Compared with Yin’s study [5], a small amount of Sc and Zr addition obtained a similar strengthening effect.

## 4. Discussion

### 4.1. The Effect of Addition of Sc and Zr on the As-Cast Microstructure of AA5182

In pure Al or binary alloys, the effect of grain refining can be obtained when the content of Sc is above 0.55 wt.% (eutectic composition) [19,23]. A small amount of Sc in the case of multi-element alloys, which does not exceed Al-Sc eutectic composition, can still achieve the same end [24,25]. After adding Sc for microalloying, based on research from Fuller et al. [26], the addition of 0.1 wt.% Zr as a dispersoid-forming element has a greater effect. As Figure 1 shows, when 0.1 wt.% Sc and Zr was added to 5182 alloy, the average ingot grain size of 0.4 mm was significantly smaller than that of the 5182 alloy. The results indicate that the microalloying of 0.1% Sc and 0.1% Zr can significantly refine the as-cast microstructure of 5182 alloy.

In the studies of Zhang and Seidman [22,27], the grain size of pure aluminum was 1~2 mm when 0.3% Sc was added. The aim of refining pure aluminum can only be achieved when 0.7% Sc is added. By adding 0.5% Sc and 0.15% Zr to pure aluminum, the grain size of the ingot was reduced to 0.12 mm.

The effect of grain refinement of Sc is more obvious in multi-element or commercial alloys than in pure metals. In the Al-5Mg alloy, with the addition of Sc increasing from 0.2% to 0.6%, the grain size changed from 0.37 mm to 0.072 mm. In Singh’s study [28], for AA8090, the addition of 0.43% Sc reduced the grain size from 0.1 mm to 0.03 mm. In this study, the addition of 0.1% Sc and 0.1% Zr in AA5182 also showed evidence of a refining effect. The improvement in the grain refinement effect is explained by the presence of Mg and other components. The solid solubility of Sc and Zr in Al matrix was reduced, which led to more primary Al_3_(Sc_x_Zr_1−x_) dispersoids being precipitated [29,30]. The nucleation rate increased during casting, which made the addition of 0.1% Sc and 0.1% effective for refinement of AA5182 grains.

### 4.2. Effect of Sc Addition and Zr on the Recrystallization Microstructure of 5182 Alloy

By adding Sc and Zr, the recrystallization was inhibited, providing conditions for the production of various semi-finished products with a stable non-recrystallized structure. In the processing of semi-finished products, the strengthening effect obtained by adding Sc and Zr mainly comes from the retention of deformed structure and the Sc-containing dispersoids that are coherent to the matrix. Optimal Sc content to ensure that the semi-finished product maintains a non-recrystallized structure after heat treatment depends on the alloy system. For 5xxx Al alloys, which are easy recrystallized, more Sc (0.25~0.30%) is required than for 7xxx Al alloys. In 7xxx Al alloys, Sc content in the range of 0.15~0.20 wt.% ensures the retention of the deformed structure of the quenched semi-product [23]. Unlike the AA5182 alloy, which is fully recrystallized after annealing at 300 °C for 2 h, the recrystallization rate of 5182-Sc-Zr is as low as 15.7%. After annealing at 500 °C for 2 h, some grains of the 5182 alloy grew abnormally, and the recrystallization rate of 5182-Sc-Zr alloy was 30.1%, maintaining a relatively low recrystallization rate. The results confirm that the combined addition of 0.1% Sc and 0.1% Zr can effectively prevent the microstructure of 5182 alloy from recrystallization. In Jia’s research [31], Al-Sc-Zr alloy with 0.15% Sc did not completely recrystallize when annealing below 600 °C, and the improvement of recrystallization resistance was related to the effective dislocation and grain boundary pinning by Al_3_(Sc_x_Zr_1−x_). The mismatch between Al_3_(Sc_x_Zr_1−x_) and the Al matrix is expected to increase the resistance of the dislocation motion [11,22,32]. This allows the dispersoids to interact strongly with the dislocations and affect their motion, thus having an effect on the pinned dislocations of the alloy. In this study, as shown in Figure 4, dislocations accumulated around the dispersoids, demonstrating that the Sc- and Zr-containing dispersoids can inhibit movement of dislocations effectively, resulting in the increase of recrystallization resistance of 5182 alloy.

### 4.3. Strengthening Mechanisms of 5182-Sc-Zr Alloy

Starting from the refinement of the ingot grains, the addition of Sc and Zr can prevent deformed microstructure from recrystallization during the annealing process and can inhibit the movement of dislocations during deformation, which leads to higher strength and better ductility of 5182-Sc-Zr. As shown in Figure 6, the strength of both cold rolled and annealed 5182-Sc-Zr alloy were higher than those of 5182 alloy. Orowan strengthening and grain boundary strengthening are the main reasons for the increase of 5182-Sc-Zr alloy strength. First, Sc- and Zr-bearing dispersoids formed in the homogenization process will interact with the dislocations and hinder the dislocation movement in the deformation process. In the deformation process, the movement of dislocations near the fine and dispersed Al_3_(Sc_x_Zr_1−x_) is impeded. According to Marquis’s study [33], when the size of the dispersoid is larger than 2.4 nm, the dislocation motion mechanism should be a bypass mechanism, resulting in Orowan strengthening. Then, the grain size of 5182-Sc-Zr alloy should range from 8.5 μm to 15.8 μm after annealing, resulting in a grain boundary strengthening effect. Finally, as shown in Figure 4, the movement and annihilation of dislocations could be inhibited during annealing, which improved the strength of the 5182-Sc-Zr alloy during a tensile test. Because the contribution of various reinforcement mechanisms is uncertain, in this study, the contribution of Orowan strengthening and grain boundary strengthening was calculated. With regard to the dislocation strengthening, the statistics of dislocation density are not precise, so dislocation strengthening was not calculated.

#### 4.3.1. Orowan Strengthening of the Dispersoids Containing Sc and Zr

The interaction between the dispersoids and dislocations noted in Figure 4 was consistent with Orowan strengthening. The thickness of the study area was assumed to be approximately 80 nm, which was an estimate from TEM sample preparation [34]. The microstructure characteristics of Al_3_(Sc_x_Zr_1−x_) particles in the annealing alloys are shown in Table 4.

The increase in yield strength due to Orowan strengthening ∆σ_*Or*_ can be calculated using the following formula [17,34]:

(1) $$\Delta \sigma_{Or}=K_{4}M{\left(1-\upsilon \right)}^{-0.5}\left(\frac{\mathrm{Gb}}{\lambda }\right)\ln\left(\frac{d_{s}}{b}\right)$$

(2) $$d_{s}=\frac{{\pi d}_{m}}{4}.$$

(3) $$\lambda =\left[\frac{1}{2}{\left(\frac{2\pi }{3f_{v}}\right)}^{0.5}-1\right]\frac{{\pi d}_{m}}{4}.$$

In Formula (1), M is the Taylor factor, υ is the Poisson’s ratio of Al matrix, G is the material shear modulus, and b is the magnitude of Al matrix Burgers vector. K_4_ is a constant and is determined based on the size and distribution of the precipitated phase. d_s_ is the average size of the precipitated phases, and λ corresponds to effective particle spacing. The Orowan strengthening formula shows that the strengthening is mainly related to the diameter and distribution of second phase particles. The values in Table 4 and the following values (K_4_ = 0.127, υ = 0.331, M = 3.1, G = 26 GN m^−2^, b = 0.286 nm) were used to calculate the Orowan strengthening of the Sc- and Zr-containing alloy. After annealing at 230 °C, 250 °C, 300 °C, 400 °C and 500 °C, the increase in yield strength brought by the dispersoids was 51.1 MPa, 46.9 Mpa, 45.9 Mpa, 44.9 Mpa and 35.2 Mpa. The strengthening effect of Orowan is influenced by the size of the second phase [19]. In a study by Vo [35], with the increase of precipitate size, the Orowan strengthening decreased from 97 MPa to 62 MPa. It was concluded that Orowan strengthening effect decreased with the increase of Al_3_(Sc_x_Zr_1−x_) size in the 5182-Sc-Zr alloy.

#### 4.3.2. Grain Boundary Strengthening of 5182 Alloy Containing Sc and Zr

Grain boundary strengthening refers to improving the strength of an alloy through the refinement of grain size. The relationship between grain size and strengthening conforms to the Hall–Petch formula shown below:

(4) $$\sigma_{\mathrm{gb}}=k_{y}d^{-1/2}.$$

In this formula, k_y_ is a parameter describing the relative strengthening contribution of the grain boundary. To estimate the strengthening provided by grain boundaries, 170 MPa * μm^1/2^ is used [36]. Compared with the 5182-based alloy, the grain size of 5182-Sc-Zr alloy was smaller, and there was a different value of YS between the two alloys, recorded as Δσ_gb_, as shown in the following formula.

(5) $${\Delta \sigma }_{\mathrm{gb}}=k\left(d_{\mathrm{Sc}-\mathrm{containing}}^{-\frac{1}{2}}-d_{\mathrm{Sc}-\mathrm{free}}^{-\frac{1}{2}}\right).$$

Substitute the data in Table 5 to obtain the grain boundary strengthening of annealed sheets. For the 5182 alloy, calculations demonstrate that the grain boundary strengthening obtained after annealing at 230 °C, 250 °C, 300 °C, 400 °C and 500 °C was 45.76 MPa, 45.43 MPa, 39.52 MPa, 37 MPa and 11.4 MPa, respectively. The 5182-Sc-Zr alloys have a higher grain boundary strengthening effect, which was 58.3 MPa, 51 MPa, 50.6 MPa, 48.67 MPa, 42.77 MPa. The Δσ_gb_ of the 5182 alloy and 5182-Sc-Zr alloy is shown in Table 6.

The contributions of Orowan strengthening and grain boundary strengthening to the yield strength of Sc- and Zr-containing alloys are recorded in Table 6. Comparing the two results of Orowan strengthening and grain boundary strengthening, it can be seen that the contribution of Orowan strengthening is higher than that of grain boundary strengthening, which proves that the strengthening effect of the 5182-Sc-Zr alloy is mainly a contribution from Orowan strengthening. In addition, in this study, contributions from Orowan strengthening and grain boundary strengthening were not enough to fully explain the strength improvement of the 5182-Sc-Zr alloy; the additional strength improvement was mainly contributed by substructure strengthening or dislocation strengthening [37].

## 5. Conclusions

In this work, AA5182 microalloyed by Sc and Zr was investigated. After adding a small amount of Sc and Zr to 5182 alloy, the dispersoids containing Sc and Zr formed after homogenization. These dispersoids led to a reduction in ingots grain size and changes in the microstructure and mechanical properties of the alloy after annealing. By studying the microstructure evolution and mechanical properties of Sc and Zr containing AA5182, the following conclusions can be drawn:

1. Adding 0.1% Sc and 0.1% Zr to commercial 5182 alloy can effectively refine ingot grains. The average grain size of 5182 alloy ingot was 0.7 mm (±0.4 mm). After adding Sc and Zr, the grain size of ingot was reduced to 0.4 mm (±0.2 mm).
2. The Al_3_(Sc_x_Zr_1−x_) dispersoids can prevent a 5182-Sc-Zr alloy from recrystallization during the annealing process. Even when annealed at 500 °C for 2 h, the recrystallization rate of the 5182-Sc-Zr alloy was 30.1%, while the 5182 alloy had already completely recrystallized after being annealed at 300 °C for 2 h.
3. For as-cold rolled sheets, the YS of 5182 alloy and 5182-Sc-Zr alloy was 283.0 MPa and 371.7 MPa, respectively. The yield strength of the 5182-Sc-Zr alloy changed from 371 MPa to 187 MPa with increasing annealing temperature, which was higher than the YS of 5182 alloy, which changed from 283 MPa to 94.2 MPa.
4. Both Orowan strengthening and fine grain strengthening were calculated to contribute to the strengthening of the 5182-Sc-Zr alloy. The contribution of Orowan strengthening to as-annealed 5182-Sc-Zr was higher than that of grain boundary strengthening.

## Figures and Tables

**Table 1:** Chemical composition of as-cast samples (wt.%).

Alloys	Mg	Mn	Cu	Ti	Fe	Si	Zr	Sc
5182 Alloy	4.53	0.22	0.0003	0.005	0.175	0.068	-	-
5182-Sc-Zr Alloy	4.44	0.22	0.0003	0.005	0.168	0.67	0.123	0.1

**Table 2:** Recrystallization rate of annealed samples.

230 °C		250 °C		300 °C		400 °C		500 °C	
AA5182	AA5182-Sc-Zr	AA5182	AA5182-Sc-Zr	AA5182	AA5182-Sc-Zr	AA5182	AA5182-Sc-Zr	AA5182	AA5182-Sc-Zr
11.8	10.3	18.9	10.8	92.9	15.7	91.0	24.4	-	30.1

**Table 3:** Tensile properties of cold rolled and annealed samples.

Tensile Properties	Cold Rolling 70%		Annealing 230 °C 2 h		Annealing 250 °C 2 h		Annealing 300 °C 2 h		Annealing 400 °C 2 h		Annealing 500 °C 2 h	
	5182 Alloy	5182-Sc-Zr Alloy	5182 Alloy	5182-Sc-Zr Alloy	5182 Alloy	5182-Sc-Zr Alloy	5182 Alloy	5182-Sc-Zr Alloy	5182 Alloy	5182-Sc-Zr Alloy	5182 Alloy	5182-Sc-Zr Alloy
YS (MPa)	283.0	371.7	267.8	360.3	237.1	277.7	147.7	247.8	139.2	211.8	94.6	187.4
TS (MPa)	378.8	418.8	346.5	409.5	334.1	366.4	290.1	354.2	287.7	343.9	236.8	329.9
n	0.150	0.0787	0.151	0.0831	0.193	0.178	0.329	0.204	0.349	0.238	0.370	0.314

**Table 4:** Microstructure characteristics of Al_3_(Sc_x_Zr_1−x_) particles.

Microstructure Characteristics	Al_3_(Sc_x_Zr_1−x_)				
	230 °C	250 °C	300 °C	400 °C	500 °C
d_m_ (nm)	15.2 ± 5.6	17.1 ± 4.6	17.6 ± 4.7	18.1 ± 1.1	25 ± 7.6
*f*_v_	10^−3^	10^−3^	10^−3^	10^−3^	10^−3^

**Table 5:** Grain size of annealed samples.

Grain Size		230 °C	250 °C	300 °C	400 °C	500 °C
d/μm	5182 Alloy	13.8	14.0	18.5	21.1	220.9
	5182-Sc-Zr Alloy	8.5	11.1	11.3	12.2	15.8

**Table 6:** Orowan strengthening and grain boundary strengthening.

Strengthening Type	Annealed at 230 °C	Annealed at 250 °C	Annealed at 300 °C	Annealed at 400 °C	Annealed at 500 °C
Δσ_Or_ (MPa)	51.1	46.9	45.9	44.9	35.2
Δσ_gb_ (MPa)	12.6	5.6	11.1	11.7	31.4

## Data Availability

Not applicable.
